# Enrofloxacinium picrate

**DOI:** 10.1107/S160053681100170X

**Published:** 2011-01-22

**Authors:** Jerry P. Jasinski, Ray J. Butcher, M. S. Siddegowda, H. S. Yathirajan, B. P. Siddaraju

**Affiliations:** a Department of Chemistry, Keene State College, 229 Main Street, Keene, NH 03435-2001, USA; b Department of Chemistry, Howard University, 525 College Street NW, Washington, DC 20059, USA; c Department of Studies in Chemistry, University of Mysore, Manasagangotri, Mysore 570 006, India

## Abstract

There is one cation–anion pair in the asymmetric unit of the title compound [systematic name: 4-(3-carb­oxy-6-fluoro-4-oxo-1,4-dihydroquinolin-7-yl)-1-ethyl­piperazin-1-ium 2,4,6-tri­nitro­phenolate], C_19_H_23_FN_3_O_3_^+^·C_6_H_2_N_3_O_7_^−^. The six-membered piperazine group in the cation adopts a slightly distorted chair conformation and contains a protonated N atom. The dihedral angles between the mean planes of the cyclo­propyl and piperazine rings in the cation with the 10-atom ring system of the quinolone group are 48.1 (1) and 69.9 (5)°, respectively. The picrate anion inter­acts with the protonated N atom of an adjacent cation through a bifurcated N—H⋯O three-center hydrogen bond, forming an *R*_1_^2^(6) ring motif. Furthermore, there is an intra­molecular O—H⋯O hydrogen bond. The dihedral angle between the mean planes of the anion benzene and cation piperizine, quinoline and cyclo­propyl rings are 61.3 (6), 31.1 (4) and 70.4 (9)°, respectively. The mean planes of the two *o*-NO_2_ and single *p*-NO_2_ groups in the picrate anion are twisted by 6.7 (6), 38.3 (9) and 12.8 (7)° with respect to the mean plane of the benzene ring. Strong N—H⋯O and weak inter­molecular C—H⋯O hydrogen bonds in concert with weak π–π stacking inter­actions [centroid–centroid distances = 3.5785 (13), 3.7451 (12) and 3.6587 (13) Å] dominate the crystal packing.

## Related literature

For background to fluoro­quinolones, see: Bhanot *et al.* (2001 ▶); Scholar (2003 ▶). For related structures, see: Hu & Yu, (2005 ▶); Jasinski *et al.* (2009 ▶, 2010*a* ▶, 2010*b* ▶); Recillas-Mota *et al.* (2007 ▶); Sun *et al.* (2004 ▶); Wang *et al.* (2005 ▶); Zou *et al.* (2005 ▶). For puckering parameters, see: Cremer & Pople (1975 ▶). For standard bond lengths, see: Allen *et al.* (1987) ▶.
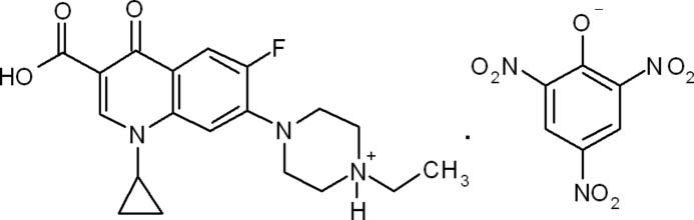


## Experimental

### 

#### Crystal data

C_19_H_23_FN_3_O_3_^+^·C_6_H_2_N_3_O_7_^−^
*M* *_r_* = 588.51
Triclinic, 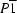

*a* = 7.2111 (7) Å
*b* = 12.5766 (7) Å
*c* = 16.2362 (4) Å
α = 105.556 (2)°
β = 96.367 (6)°
γ = 96.223 (7)°
*V* = 1395.04 (16) Å^3^
*Z* = 2
Cu *K*α radiation
μ = 0.98 mm^−1^
*T* = 295 K
0.44 × 0.31 × 0.12 mm

#### Data collection

Oxford Diffraction Xcalibur Ruby Gemini diffractometer
Absorption correction: multi-scan (*CrysAlis RED*; Oxford Diffraction, 2007 ▶) *T*_min_ = 0.896, *T*_max_ = 1.000
9440 measured reflections
5437 independent reflections
3425 reflections with *I* > 2σ(*I*)
*R*_int_ = 0.032

#### Refinement

*R*[*F* ^2^ > 2σ(*F* ^2^)] = 0.055
*wR*(*F* ^2^) = 0.177
*S* = 1.00
5437 reflections
382 parameters
H-atom parameters constrained
Δρ_max_ = 0.20 e Å^−3^
Δρ_min_ = −0.20 e Å^−3^

### 

Data collection: *CrysAlis PRO* (Oxford Diffraction, 2007 ▶); cell refinement: *CrysAlis PRO*; data reduction: *CrysAlis RED* (Oxford Diffraction, 2007 ▶); program(s) used to solve structure: *SHELXS97* (Sheldrick, 2008 ▶); program(s) used to refine structure: *SHELXL97* (Sheldrick, 2008 ▶); molecular graphics: *SHELXTL* (Sheldrick, 2008 ▶); software used to prepare material for publication: *SHELXTL*.

## Supplementary Material

Crystal structure: contains datablocks global, I. DOI: 10.1107/S160053681100170X/bt5451sup1.cif

Structure factors: contains datablocks I. DOI: 10.1107/S160053681100170X/bt5451Isup2.hkl

Additional supplementary materials:  crystallographic information; 3D view; checkCIF report

## Figures and Tables

**Table 1:** Hydrogen-bond geometry (Å, °)

*D*—H⋯*A*	*D*—H	H⋯*A*	*D*⋯*A*	*D*—H⋯*A*
O2—H2⋯O3	0.82	1.78	2.536 (3)	151
N3—H3*A*⋯O1*A*	0.91	1.87	2.724 (3)	155
N3—H3*A*⋯O7*A*	0.91	2.38	3.024 (3)	128
C11—H11*A*⋯O3^i^	0.98	2.55	3.385 (3)	144
C15—H15*B*⋯O1^ii^	0.97	2.35	3.312 (3)	169
C17—H17*B*⋯O3*A*^iii^	0.97	2.56	3.458 (4)	154
C3*A*—H3*AA*⋯O3^iv^	0.93	2.55	3.331 (3)	142
C9—H9*A*⋯O4*A*^v^	0.93	2.58	3.495 (3)	170
C14—H14*B*⋯O5*A*^vi^	0.97	2.60	3.517 (4)	157
C18—H18*A*⋯O5*A*^vii^	0.97	2.50	3.451 (5)	167

Symmetry codes: (i) 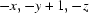; (ii) 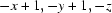; (iii) 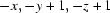; (iv) 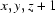; (v) 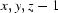; (vi) 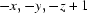; (vii) 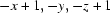.

**Fig. 1.**
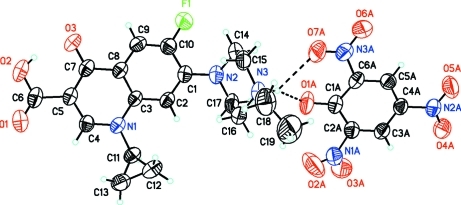
Molecular structure of the title compound showing the atom labeling scheme and 50% probability displacement ellipsoids. Dashed lines indicate a bifurcated N—H···O intermolecular, three-centered hydrogen bond formed between the protonated N atom from the enrofloxacin cation and the picrate anion providing a R12(6) ring motif.

**Fig. 2.**
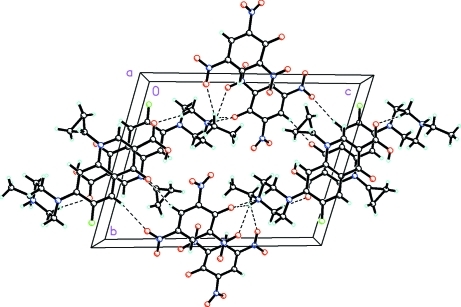
Packing diagram of the title compound viewed down the a axis. Dashed lines indicate N—H···O hydrogen bonds and weak C—H···O intermolecular interactions creating a 2-D network structure along 011.

## supplementary crystallographic information

### Comment

Enrofloxacin is a fluoroquinolone antibiotic and is a synthetic chemotherapeutic agent from the class of the fluoroquinolone carboxylic acid derivatives. It is sold by the Bayer Corporation under the trade name Baytril and has antibacterial activity against a broad spectrum of Gram-negative and Gram-positive bacteria.  Its mechanism of action is not thoroughly understood, but it is believed to act by inhibiting bacterial DNA gyrase (a type-II topoisomerase), thereby preventing DNA supercoiling and DNA synthesis.  The chemical and biological aspects of fluoroquinolones is described (Bhanot *et al.*, 2001; Scholar, 2003). The crystal structure of norfloxacin hydrochloride (Zou *et al.*, 2005) and norfloxacin methanol solvate (Wang *et al.*, 2005) have already been reported.  The crystal structure of a copper complex of enrofloxacin (Recillas-Mota *et al.*, 2007), norfloxacin picrate (Hu & Yu, 2005) and 2-hydroxyethanaminium enrofloxacinate (Sun *et al.*, 2004) are reported.  Recently, the crystal structures of propiverine picrate (Jasinski *et al.*, 2009), imatinibium dipicrate (Jasinski *et al.*, 2010*a*) and chlorimipraminium picrate (Jasinski *et al.*, 2010*b*) have been reported.  In continuation of our work on picrates of biologically active compounds, this paper reports the crystal structure of  C_19_H_22_FN_3_O_3_^+^ . C_6_H_2_N_3_O_7_^-^ obtained by the interaction of picric acid and enrofloxacin.

In the crystal structure of the title compound, (I), there is one cation-anion pair in the asymmetric unit (Fig. 1).  One N atom in the 6-membered piperazine ring (N2/C14/C15/N3/C16/C17) in the enrofloxacinium cation is protonated which adopts a slightly distorted chair conformation with puckering parameters Q, θ and φ of 0.563 (3)A%, 4.0 (3)° and 358.0 (5)° (Cremer & Pople, 1975).  The dihedral angles between the mean planes of the cyclopropyl and piperazine rings with the 10-atom ring system of the quinolone group are 48.1 (1)° and 69.9 (5)°, respectively.  The picrate anion interacts with the protonated N atom of an adjacent cation through a bifurcated N—H···O three-center hydrogen bond forming a R_1_^2^(6) ring motif. The dihedral angle between the mean planes of the anion benzene and cation piperizine, quinoline and cyclopropyl rings are 61.3 (6)°, 31.1 (4)° and 70.4 (9)°, respectively.  The mean planes of the two *o*-NO_2_ and single *p*-NO_2_ groups in the picrate anion are twisted by 6.7 (6)°, 38.3 (9)° and 12.8 (7)° with respect to the mean planes of the 6-membered benzene ring. Bond distances and angles are in normal ranges (Allen *et al.*, 1987).  Strong N—H···O and weak intermolecular C—H···O hydrogen bonds in concert with weak π–π stacking interactions (Table 2) dominate the crystal packing creating a 2-D network structure along 011 (Fig. 2).

### Experimental

Enrofloxacin (3.59 g, 0.1 mol) and picric acid (2.99 g, 0.1 mol) were dissolved in a mixture of acetonitrile and dimethyl sulfoxide (80:20 *v*/*v*). The solution was stirred for 15 min over a heating magnetic stirrer at 333 K. The resulting solution was kept aside at room temperature. After few days, X-ray quality crystals of the title compound were grown by slow evaporation (m.p.: 490 – 493 K).

### Refinement

All   H atoms were  refined using the riding model with Atom—H lengths of 0.93 & 0.98Å (CH), 0.97Å (CH_2_), 0.96Å (CH_3_), 0.91Å (NH) or 0.82 (OH). Isotropic displacement parameters for these atoms were set to 1.20 times (NH), 1.19–1.20 (CH, CH_2_) or 1.49 (CH_3_, OH) times *U*_eq_ of the parent atom.

### Crystal data

**Table:**

C_19_H_23_FN_3_O_3_^+^·C_6_H_2_N_3_O_7_^−^	*Z* = 2
*M**_r_* = 588.51	*F*(000) = 612
Triclinic, *P*1	*D*_x_ = 1.401 Mg m^−^^3^
Hall symbol: -P 1	Cu *K*α radiation, λ = 1.54178 Å
*a* = 7.2111 (7) Å	Cell parameters from 2958 reflections
*b* = 12.5766 (7) Å	θ = 5.3–73.4°
*c* = 16.2362 (4) Å	µ = 0.98 mm^−^^1^
α = 105.556 (2)°	*T* = 295 K
β = 96.367 (6)°	Plate, pale yellow
γ = 96.223 (7)°	0.44 × 0.31 × 0.12 mm
*V* = 1395.04 (16) Å^3^	

### Data collection

**Table:**

Oxford Diffraction Xcalibur Ruby Gemini diffractometer	5437 independent reflections
Radiation source: Enhance (Cu) X-ray Source	3425 reflections with *I* > 2σ(*I*)
graphite	*R*_int_ = 0.032
Detector resolution: 10.5081 pixels mm^-1^	θ_max_ = 73.6°, θ_min_ = 5.3°
ω scans	*h* = −5→8
Absorption correction: multi-scan (*CrysAlis RED*; Oxford Diffraction, 2007)	*k* = −15→14
*T*_min_ = 0.896, *T*_max_ = 1.000	*l* = −20→20
9440 measured reflections	

### Refinement

**Table:**

Refinement on *F*^2^	Secondary atom site location: difference Fourier map
Least-squares matrix: full	Hydrogen site location: inferred from neighbouring sites
*R*[*F*^2^ > 2σ(*F*^2^)] = 0.055	H-atom parameters constrained
*wR*(*F*^2^) = 0.177	*w* = 1/[σ^2^(*F*_o_^2^) + (0.0924*P*)^2^] where *P* = (*F*_o_^2^ + 2*F*_c_^2^)/3
*S* = 1.00	(Δ/σ)_max_ < 0.001
5437 reflections	Δρ_max_ = 0.20 e Å^−^^3^
382 parameters	Δρ_min_ = −0.20 e Å^−^^3^
0 restraints	Extinction correction: *SHELXL97* (Sheldrick, 2008), Fc^*^=kFc[1+0.001xFc^2^λ^3^/sin(2θ)]^-1/4^
Primary atom site location: structure-invariant direct methods	Extinction coefficient: 0.0007 (4)

### Special details

**Table:**

Geometry. All e.s.d.'s (except the e.s.d. in the dihedral angle between two l.s. planes) are estimated using the full covariance matrix. The cell e.s.d.'s are taken into account individually in the estimation of e.s.d.'s in distances, angles and torsion angles; correlations between e.s.d.'s in cell parameters are only used when they are defined by crystal symmetry. An approximate (isotropic) treatment of cell e.s.d.'s is used for estimating e.s.d.'s involving l.s. planes.
Refinement. Refinement of *F*^2^ against ALL reflections. The weighted *R*-factor *wR* and goodness of fit *S* are based on *F*^2^, conventional *R*-factors *R* are based on *F*, with *F* set to zero for negative *F*^2^. The threshold expression of *F*^2^ > σ(*F*^2^) is used only for calculating *R*-factors(gt) *etc*. and is not relevant to the choice of reflections for refinement. *R*-factors based on *F*^2^ are statistically about twice as large as those based on *F*, and *R*- factors based on ALL data will be even larger.

### Fractional atomic coordinates and isotropic or equivalent isotropic displacement parameters (Å^2^)

**Table:**

	*x*	*y*	*z*	*U*_iso_*/*U*_eq_	
F1	0.0392 (3)	0.14397 (14)	0.02905 (11)	0.0785 (5)	
O1	0.4038 (4)	0.7253 (2)	−0.10088 (16)	0.0920 (8)	
O2	0.3085 (3)	0.5648 (2)	−0.19916 (12)	0.0747 (6)	
H2	0.2552	0.5031	−0.2008	0.112*	
O3	0.2077 (3)	0.39341 (17)	−0.15203 (11)	0.0611 (5)	
N1	0.2699 (3)	0.59410 (17)	0.09706 (12)	0.0456 (5)	
N2	0.0978 (3)	0.27232 (19)	0.20117 (14)	0.0580 (6)	
N3	0.3999 (3)	0.2675 (2)	0.33307 (14)	0.0591 (6)	
H3A	0.3366	0.2332	0.3665	0.071*	
C1	0.1315 (3)	0.3205 (2)	0.13528 (16)	0.0490 (6)	
C2	0.1818 (3)	0.4344 (2)	0.14892 (15)	0.0460 (5)	
H2A	0.1934	0.4819	0.2046	0.055*	
C3	0.2153 (3)	0.47948 (19)	0.08107 (13)	0.0405 (5)	
C4	0.3062 (3)	0.6357 (2)	0.03193 (15)	0.0465 (5)	
H4A	0.3420	0.7123	0.0444	0.056*	
C5	0.2938 (3)	0.5723 (2)	−0.05229 (15)	0.0469 (5)	
C6	0.3401 (4)	0.6293 (3)	−0.11816 (18)	0.0617 (7)	
C7	0.2321 (3)	0.4553 (2)	−0.07435 (14)	0.0465 (5)	
C8	0.1951 (3)	0.4099 (2)	−0.00384 (14)	0.0448 (5)	
C9	0.1368 (3)	0.2958 (2)	−0.01881 (16)	0.0510 (6)	
H9A	0.1199	0.2484	−0.0748	0.061*	
C10	0.1051 (4)	0.2543 (2)	0.04762 (17)	0.0553 (6)	
C11	0.2678 (4)	0.6702 (2)	0.18217 (16)	0.0519 (6)	
H11A	0.1420	0.6815	0.1971	0.062*	
C12	0.4138 (4)	0.6777 (3)	0.25637 (18)	0.0645 (7)	
H12A	0.3750	0.6903	0.3131	0.077*	
H12B	0.5118	0.6306	0.2463	0.077*	
C13	0.4137 (5)	0.7695 (3)	0.2158 (2)	0.0735 (8)	
H13A	0.5119	0.7788	0.1810	0.088*	
H13B	0.3752	0.8384	0.2478	0.088*	
C14	0.1708 (5)	0.1684 (3)	0.2041 (2)	0.0691 (8)	
H14A	0.1588	0.1193	0.1458	0.083*	
H14B	0.0958	0.1309	0.2368	0.083*	
C15	0.3727 (4)	0.1898 (3)	0.24448 (18)	0.0639 (7)	
H15A	0.4143	0.1198	0.2470	0.077*	
H15B	0.4494	0.2213	0.2090	0.077*	
C16	0.3177 (5)	0.3719 (2)	0.33310 (18)	0.0650 (7)	
H16A	0.3916	0.4147	0.3034	0.078*	
H16B	0.3222	0.4172	0.3921	0.078*	
C17	0.1147 (4)	0.3434 (3)	0.28845 (17)	0.0612 (7)	
H17A	0.0393	0.3063	0.3213	0.073*	
H17B	0.0651	0.4118	0.2874	0.073*	
C18	0.6053 (5)	0.2905 (4)	0.3705 (3)	0.0917 (11)	
H18A	0.6582	0.2213	0.3560	0.110*	
H18B	0.6707	0.3413	0.3441	0.110*	
C19	0.6391 (7)	0.3390 (4)	0.4650 (3)	0.1301 (18)	
H19A	0.7709	0.3660	0.4836	0.195*	
H19B	0.6006	0.2830	0.4923	0.195*	
H19C	0.5677	0.3997	0.4807	0.195*	
O1A	0.1749 (3)	0.2238 (2)	0.44767 (13)	0.0791 (7)	
O2A	0.1956 (6)	0.4178 (2)	0.5795 (2)	0.1384 (15)	
O3A	−0.0251 (4)	0.3840 (2)	0.64871 (17)	0.0934 (8)	
O4A	0.0864 (4)	0.0906 (2)	0.78322 (15)	0.0947 (8)	
O5A	0.2090 (4)	−0.0501 (2)	0.71552 (16)	0.0919 (8)	
O6A	0.3078 (3)	−0.09139 (19)	0.42732 (14)	0.0761 (6)	
O7A	0.3468 (4)	0.0487 (2)	0.37754 (15)	0.0939 (8)	
N1A	0.0996 (4)	0.3558 (2)	0.60751 (17)	0.0724 (7)	
N2A	0.1542 (4)	0.0399 (2)	0.72150 (14)	0.0640 (6)	
N3A	0.2989 (3)	0.0068 (2)	0.43254 (14)	0.0594 (6)	
C1A	0.1855 (3)	0.1834 (2)	0.50984 (16)	0.0528 (6)	
C2A	0.1375 (4)	0.2406 (2)	0.59322 (17)	0.0531 (6)	
C3A	0.1247 (4)	0.1958 (2)	0.65942 (16)	0.0526 (6)	
H3AA	0.0879	0.2362	0.7103	0.063*	
C4A	0.1673 (3)	0.0878 (2)	0.65059 (15)	0.0494 (6)	
C5A	0.2252 (3)	0.0292 (2)	0.57639 (16)	0.0489 (5)	
H5AA	0.2573	−0.0416	0.5717	0.059*	
C6A	0.2361 (3)	0.0748 (2)	0.50884 (15)	0.0486 (6)	

### Atomic displacement parameters (Å^2^)

**Table:**

	*U*^11^	*U*^22^	*U*^33^	*U*^12^	*U*^13^	*U*^23^
F1	0.1083 (14)	0.0580 (10)	0.0638 (11)	−0.0030 (9)	0.0032 (9)	0.0181 (8)
O1	0.129 (2)	0.0800 (16)	0.0781 (15)	−0.0007 (15)	0.0328 (14)	0.0403 (13)
O2	0.0854 (14)	0.1032 (17)	0.0458 (11)	0.0184 (12)	0.0220 (9)	0.0319 (10)
O3	0.0700 (11)	0.0758 (12)	0.0350 (9)	0.0115 (9)	0.0124 (8)	0.0093 (8)
N1	0.0441 (10)	0.0565 (12)	0.0375 (10)	0.0120 (9)	0.0084 (8)	0.0128 (8)
N2	0.0635 (13)	0.0660 (14)	0.0527 (12)	0.0125 (11)	0.0111 (10)	0.0286 (11)
N3	0.0636 (13)	0.0714 (15)	0.0543 (13)	0.0129 (11)	0.0117 (10)	0.0358 (11)
C1	0.0479 (12)	0.0589 (15)	0.0458 (13)	0.0112 (11)	0.0087 (10)	0.0224 (11)
C2	0.0479 (12)	0.0560 (14)	0.0356 (11)	0.0150 (10)	0.0049 (9)	0.0130 (10)
C3	0.0372 (10)	0.0521 (13)	0.0344 (10)	0.0139 (9)	0.0058 (8)	0.0131 (9)
C4	0.0425 (11)	0.0555 (14)	0.0456 (13)	0.0111 (10)	0.0099 (9)	0.0182 (10)
C5	0.0433 (12)	0.0630 (15)	0.0403 (12)	0.0147 (11)	0.0100 (9)	0.0200 (11)
C6	0.0618 (16)	0.084 (2)	0.0526 (15)	0.0217 (15)	0.0219 (12)	0.0319 (14)
C7	0.0368 (11)	0.0665 (15)	0.0392 (12)	0.0155 (10)	0.0083 (9)	0.0157 (11)
C8	0.0400 (11)	0.0586 (14)	0.0383 (11)	0.0161 (10)	0.0055 (9)	0.0147 (10)
C9	0.0544 (13)	0.0554 (14)	0.0408 (12)	0.0149 (11)	0.0044 (10)	0.0077 (10)
C10	0.0579 (14)	0.0553 (15)	0.0515 (14)	0.0071 (12)	0.0039 (11)	0.0156 (11)
C11	0.0520 (13)	0.0588 (15)	0.0439 (13)	0.0149 (11)	0.0109 (10)	0.0087 (11)
C12	0.0591 (15)	0.0800 (19)	0.0465 (14)	0.0117 (14)	0.0044 (11)	0.0053 (13)
C13	0.087 (2)	0.0675 (19)	0.0558 (17)	−0.0051 (16)	0.0171 (15)	0.0045 (13)
C14	0.094 (2)	0.0607 (17)	0.0593 (17)	0.0093 (15)	0.0109 (15)	0.0299 (14)
C15	0.085 (2)	0.0666 (17)	0.0584 (16)	0.0319 (15)	0.0293 (14)	0.0329 (13)
C16	0.092 (2)	0.0609 (17)	0.0481 (15)	0.0189 (15)	0.0131 (13)	0.0205 (12)
C17	0.0722 (17)	0.0759 (18)	0.0541 (15)	0.0292 (14)	0.0270 (13)	0.0352 (13)
C18	0.073 (2)	0.114 (3)	0.100 (3)	0.014 (2)	−0.0005 (19)	0.054 (2)
C19	0.124 (4)	0.145 (4)	0.107 (4)	−0.007 (3)	−0.042 (3)	0.045 (3)
O1A	0.0990 (15)	0.1074 (17)	0.0575 (12)	0.0444 (13)	0.0295 (11)	0.0493 (12)
O2A	0.217 (4)	0.0739 (18)	0.162 (3)	0.035 (2)	0.101 (3)	0.061 (2)
O3A	0.1141 (19)	0.0871 (17)	0.0886 (17)	0.0479 (15)	0.0326 (15)	0.0208 (13)
O4A	0.158 (2)	0.0823 (16)	0.0574 (13)	0.0197 (15)	0.0483 (15)	0.0295 (11)
O5A	0.137 (2)	0.0827 (16)	0.0819 (16)	0.0374 (15)	0.0402 (15)	0.0491 (13)
O6A	0.0938 (15)	0.0690 (14)	0.0653 (13)	0.0195 (11)	0.0230 (11)	0.0112 (10)
O7A	0.143 (2)	0.0989 (17)	0.0629 (14)	0.0416 (16)	0.0566 (15)	0.0364 (12)
N1A	0.0965 (19)	0.0661 (16)	0.0625 (15)	0.0235 (14)	0.0164 (14)	0.0250 (12)
N2A	0.0870 (16)	0.0625 (15)	0.0475 (12)	0.0052 (12)	0.0192 (11)	0.0224 (11)
N3A	0.0624 (13)	0.0688 (16)	0.0470 (12)	0.0137 (11)	0.0110 (10)	0.0136 (11)
C1A	0.0504 (13)	0.0682 (16)	0.0464 (13)	0.0124 (12)	0.0100 (10)	0.0250 (12)
C2A	0.0553 (14)	0.0580 (15)	0.0505 (14)	0.0104 (11)	0.0113 (11)	0.0206 (11)
C3A	0.0556 (14)	0.0602 (15)	0.0410 (12)	0.0048 (11)	0.0099 (10)	0.0130 (11)
C4A	0.0541 (13)	0.0545 (14)	0.0415 (12)	0.0022 (11)	0.0105 (10)	0.0180 (10)
C5A	0.0489 (12)	0.0482 (13)	0.0495 (13)	0.0036 (10)	0.0071 (10)	0.0151 (10)
C6A	0.0468 (12)	0.0622 (15)	0.0360 (11)	0.0056 (11)	0.0072 (9)	0.0128 (10)

### Geometric parameters (Å, °)

**Table:**

F1—C10	1.358 (3)	C13—H13B	0.9700
O1—C6	1.191 (4)	C14—C15	1.493 (4)
O2—C6	1.327 (4)	C14—H14A	0.9700
O2—H2	0.8200	C14—H14B	0.9700
O3—C7	1.274 (3)	C15—H15A	0.9700
N1—C4	1.337 (3)	C15—H15B	0.9700
N1—C3	1.398 (3)	C16—C17	1.519 (4)
N1—C11	1.457 (3)	C16—H16A	0.9700
N2—C1	1.394 (3)	C16—H16B	0.9700
N2—C17	1.443 (4)	C17—H17A	0.9700
N2—C14	1.472 (4)	C17—H17B	0.9700
N3—C15	1.485 (4)	C18—C19	1.473 (6)
N3—C16	1.497 (4)	C18—H18A	0.9700
N3—C18	1.503 (4)	C18—H18B	0.9700
N3—H3A	0.9100	C19—H19A	0.9600
C1—C2	1.390 (3)	C19—H19B	0.9600
C1—C10	1.423 (4)	C19—H19C	0.9600
C2—C3	1.399 (3)	O1A—C1A	1.245 (3)
C2—H2A	0.9300	O2A—N1A	1.199 (4)
C3—C8	1.403 (3)	O3A—N1A	1.207 (3)
C4—C5	1.373 (3)	O4A—N2A	1.218 (3)
C4—H4A	0.9300	O5A—N2A	1.222 (3)
C5—C7	1.425 (4)	O6A—N3A	1.224 (3)
C5—C6	1.486 (3)	O7A—N3A	1.215 (3)
C7—C8	1.447 (3)	N1A—C2A	1.465 (4)
C8—C9	1.398 (4)	N2A—C4A	1.443 (3)
C9—C10	1.349 (4)	N3A—C6A	1.453 (3)
C9—H9A	0.9300	C1A—C6A	1.447 (4)
C11—C13	1.479 (4)	C1A—C2A	1.451 (4)
C11—C12	1.485 (4)	C2A—C3A	1.348 (3)
C11—H11A	0.9800	C3A—C4A	1.399 (4)
C12—C13	1.475 (5)	C3A—H3AA	0.9300
C12—H12A	0.9700	C4A—C5A	1.368 (3)
C12—H12B	0.9700	C5A—C6A	1.373 (3)
C13—H13A	0.9700	C5A—H5AA	0.9300
			
C6—O2—H2	109.5	N2—C14—H14A	109.3
C4—N1—C3	119.8 (2)	C15—C14—H14A	109.3
C4—N1—C11	119.3 (2)	N2—C14—H14B	109.3
C3—N1—C11	120.48 (19)	C15—C14—H14B	109.3
C1—N2—C17	118.9 (2)	H14A—C14—H14B	107.9
C1—N2—C14	120.3 (2)	N3—C15—C14	111.4 (2)
C17—N2—C14	108.4 (2)	N3—C15—H15A	109.3
C15—N3—C16	110.8 (2)	C14—C15—H15A	109.3
C15—N3—C18	110.0 (3)	N3—C15—H15B	109.3
C16—N3—C18	112.6 (3)	C14—C15—H15B	109.3
C15—N3—H3A	107.7	H15A—C15—H15B	108.0
C16—N3—H3A	107.7	N3—C16—C17	110.3 (2)
C18—N3—H3A	107.7	N3—C16—H16A	109.6
C2—C1—N2	123.5 (2)	C17—C16—H16A	109.6
C2—C1—C10	115.7 (2)	N3—C16—H16B	109.6
N2—C1—C10	120.7 (2)	C17—C16—H16B	109.6
C1—C2—C3	121.8 (2)	H16A—C16—H16B	108.1
C1—C2—H2A	119.1	N2—C17—C16	112.2 (2)
C3—C2—H2A	119.1	N2—C17—H17A	109.2
N1—C3—C2	120.5 (2)	C16—C17—H17A	109.2
N1—C3—C8	119.3 (2)	N2—C17—H17B	109.2
C2—C3—C8	120.3 (2)	C16—C17—H17B	109.2
N1—C4—C5	124.0 (2)	H17A—C17—H17B	107.9
N1—C4—H4A	118.0	C19—C18—N3	113.3 (3)
C5—C4—H4A	118.0	C19—C18—H18A	108.9
C4—C5—C7	119.5 (2)	N3—C18—H18A	108.9
C4—C5—C6	118.4 (2)	C19—C18—H18B	108.9
C7—C5—C6	122.1 (2)	N3—C18—H18B	108.9
O1—C6—O2	121.3 (3)	H18A—C18—H18B	107.7
O1—C6—C5	123.5 (3)	C18—C19—H19A	109.5
O2—C6—C5	115.2 (3)	C18—C19—H19B	109.5
O3—C7—C5	122.2 (2)	H19A—C19—H19B	109.5
O3—C7—C8	121.2 (2)	C18—C19—H19C	109.5
C5—C7—C8	116.6 (2)	H19A—C19—H19C	109.5
C9—C8—C3	118.4 (2)	H19B—C19—H19C	109.5
C9—C8—C7	120.8 (2)	O2A—N1A—O3A	123.2 (3)
C3—C8—C7	120.8 (2)	O2A—N1A—C2A	118.8 (3)
C10—C9—C8	120.2 (2)	O3A—N1A—C2A	118.0 (3)
C10—C9—H9A	119.9	O4A—N2A—O5A	123.8 (2)
C8—C9—H9A	119.9	O4A—N2A—C4A	118.1 (2)
C9—C10—F1	117.8 (2)	O5A—N2A—C4A	118.1 (2)
C9—C10—C1	123.5 (3)	O7A—N3A—O6A	121.8 (2)
F1—C10—C1	118.6 (2)	O7A—N3A—C6A	119.9 (2)
N1—C11—C13	119.6 (2)	O6A—N3A—C6A	118.2 (2)
N1—C11—C12	121.4 (2)	O1A—C1A—C6A	126.2 (2)
C13—C11—C12	59.7 (2)	O1A—C1A—C2A	122.3 (3)
N1—C11—H11A	115.0	C6A—C1A—C2A	111.4 (2)
C13—C11—H11A	115.0	C3A—C2A—C1A	124.9 (2)
C12—C11—H11A	115.0	C3A—C2A—N1A	116.8 (2)
C13—C12—C11	60.0 (2)	C1A—C2A—N1A	118.3 (2)
C13—C12—H12A	117.8	C2A—C3A—C4A	119.2 (2)
C11—C12—H12A	117.8	C2A—C3A—H3AA	120.4
C13—C12—H12B	117.8	C4A—C3A—H3AA	120.4
C11—C12—H12B	117.8	C5A—C4A—C3A	120.4 (2)
H12A—C12—H12B	114.9	C5A—C4A—N2A	120.3 (2)
C12—C13—C11	60.36 (19)	C3A—C4A—N2A	119.3 (2)
C12—C13—H13A	117.7	C4A—C5A—C6A	120.1 (2)
C11—C13—H13A	117.7	C4A—C5A—H5AA	120.0
C12—C13—H13B	117.7	C6A—C5A—H5AA	120.0
C11—C13—H13B	117.7	C5A—C6A—C1A	123.7 (2)
H13A—C13—H13B	114.9	C5A—C6A—N3A	116.4 (2)
N2—C14—C15	111.8 (2)	C1A—C6A—N3A	119.9 (2)
			
C17—N2—C1—C2	0.3 (4)	C3—N1—C11—C12	74.5 (3)
C14—N2—C1—C2	−137.6 (3)	N1—C11—C12—C13	108.3 (3)
C17—N2—C1—C10	−176.1 (2)	N1—C11—C13—C12	−111.2 (3)
C14—N2—C1—C10	46.0 (3)	C1—N2—C14—C15	82.1 (3)
N2—C1—C2—C3	179.7 (2)	C17—N2—C14—C15	−59.7 (3)
C10—C1—C2—C3	−3.8 (3)	C16—N3—C15—C14	−52.8 (3)
C4—N1—C3—C2	178.3 (2)	C18—N3—C15—C14	−177.9 (2)
C11—N1—C3—C2	−8.4 (3)	N2—C14—C15—N3	57.1 (3)
C4—N1—C3—C8	−1.5 (3)	C15—N3—C16—C17	52.0 (3)
C11—N1—C3—C8	171.75 (19)	C18—N3—C16—C17	175.7 (2)
C1—C2—C3—N1	−178.5 (2)	C1—N2—C17—C16	−82.7 (3)
C1—C2—C3—C8	1.3 (3)	C14—N2—C17—C16	59.7 (3)
C3—N1—C4—C5	−0.1 (3)	N3—C16—C17—N2	−57.0 (3)
C11—N1—C4—C5	−173.4 (2)	C15—N3—C18—C19	−163.2 (3)
N1—C4—C5—C7	2.5 (3)	C16—N3—C18—C19	72.6 (4)
N1—C4—C5—C6	−179.3 (2)	O1A—C1A—C2A—C3A	171.7 (3)
C4—C5—C6—O1	7.0 (4)	C6A—C1A—C2A—C3A	−5.5 (4)
C7—C5—C6—O1	−174.8 (3)	O1A—C1A—C2A—N1A	−8.2 (4)
C4—C5—C6—O2	−174.1 (2)	C6A—C1A—C2A—N1A	174.5 (2)
C7—C5—C6—O2	4.1 (4)	O2A—N1A—C2A—C3A	140.3 (3)
C4—C5—C7—O3	176.0 (2)	O3A—N1A—C2A—C3A	−38.2 (4)
C6—C5—C7—O3	−2.2 (3)	O2A—N1A—C2A—C1A	−39.8 (4)
C4—C5—C7—C8	−3.0 (3)	O3A—N1A—C2A—C1A	141.7 (3)
C6—C5—C7—C8	178.8 (2)	C1A—C2A—C3A—C4A	2.8 (4)
N1—C3—C8—C9	−178.78 (19)	N1A—C2A—C3A—C4A	−177.3 (2)
C2—C3—C8—C9	1.4 (3)	C2A—C3A—C4A—C5A	1.4 (4)
N1—C3—C8—C7	0.7 (3)	C2A—C3A—C4A—N2A	179.8 (2)
C2—C3—C8—C7	−179.07 (19)	O4A—N2A—C4A—C5A	−173.7 (3)
O3—C7—C8—C9	2.0 (3)	O5A—N2A—C4A—C5A	5.2 (4)
C5—C7—C8—C9	−179.0 (2)	O4A—N2A—C4A—C3A	7.9 (4)
O3—C7—C8—C3	−177.5 (2)	O5A—N2A—C4A—C3A	−173.2 (3)
C5—C7—C8—C3	1.5 (3)	C3A—C4A—C5A—C6A	−2.1 (4)
C3—C8—C9—C10	−1.4 (3)	N2A—C4A—C5A—C6A	179.5 (2)
C7—C8—C9—C10	179.1 (2)	C4A—C5A—C6A—C1A	−1.3 (4)
C8—C9—C10—F1	176.6 (2)	C4A—C5A—C6A—N3A	−180.0 (2)
C8—C9—C10—C1	−1.4 (4)	O1A—C1A—C6A—C5A	−172.4 (3)
C2—C1—C10—C9	3.9 (4)	C2A—C1A—C6A—C5A	4.7 (3)
N2—C1—C10—C9	−179.4 (2)	O1A—C1A—C6A—N3A	6.3 (4)
C2—C1—C10—F1	−174.0 (2)	C2A—C1A—C6A—N3A	−176.6 (2)
N2—C1—C10—F1	2.6 (4)	O7A—N3A—C6A—C5A	−165.8 (3)
C4—N1—C11—C13	−41.8 (3)	O6A—N3A—C6A—C5A	11.8 (3)
C3—N1—C11—C13	145.0 (2)	O7A—N3A—C6A—C1A	15.4 (4)
C4—N1—C11—C12	−112.3 (3)	O6A—N3A—C6A—C1A	−167.0 (2)

### Hydrogen-bond geometry (Å, °)

**Table:**

*D*—H···*A*	*D*—H	H···*A*	*D*···*A*	*D*—H···*A*
O2—H2···O3	0.82	1.78	2.536 (3)	151
N3—H3A···O1A	0.91	1.87	2.724 (3)	155
N3—H3A···O7A	0.91	2.38	3.024 (3)	128
C11—H11A···O3^i^	0.98	2.55	3.385 (3)	144
C15—H15B···O1^ii^	0.97	2.35	3.312 (3)	169
C17—H17B···O3A^iii^	0.97	2.56	3.458 (4)	154
C3A—H3AA···O3^iv^	0.93	2.55	3.331 (3)	142
C9—H9A···O4A^v^	0.93	2.58	3.495 (3)	170
C14—H14B···O5A^vi^	0.97	2.60	3.517 (4)	157
C18—H18A···O5A^vii^	0.97	2.50	3.451 (5)	167

Symmetry codes: (i) −*x*, −*y*+1, −*z*; (ii) −*x*+1, −*y*+1, −*z*; (iii) −*x*, −*y*+1, −*z*+1; (iv) *x*, *y*, *z*+1; (v) *x*, *y*, *z*−1; (vi) −*x*, −*y*, −*z*+1; (vii) −*x*+1, −*y*, −*z*+1.

### Table 2 Cg···Cg π stacking interactions, Cg2 and Cg4 are the centroids of rings N1/C3/C8/C7/C5/C4 and C1/C2/C3/C8/C9/C10; [Symmetry codes: (i) -x, 1-y, -z; (ii) 1-x, 1-y, -z;]

**Table:**

*Cg*I···*Cg*J	Cg···Cg (Å)	CgI Perp (Å)	Cgj Perp (Å)	Slippage (Å)
Cg2···Cg2^i^	3.5785 (13)	-3.3834 (9)	-3.3834 (9)	1.16 (5)
Cg2···Cg2^ii^	3.7451 (12)	-3.6091 (9)	3.6090 (9)	1.00 (0)
Cg2···Cg4^ii^	3.6587 (13)	-3.3748 (9)	-3.4114 (10)	
